# Participatory Development of an Integrated, eHealth-Supported, Educational Care Pathway (Diabetes Box) for People With Type 2 Diabetes: Development and Usability Study

**DOI:** 10.2196/45055

**Published:** 2024-05-31

**Authors:** Daan Leonhard de Frel, Mariëlle A Schroijen, Jiska J Aardoom, Wesley van Gils, Sasja D Huisman, Veronica R Janssen, Anke Versluis, Maaike S Kleinsmann, Douwe E Atsma, Hanno Pijl

**Affiliations:** 1 Department of Cardiology Leiden University Medical Center Leiden Netherlands; 2 Division of Endocrinology Department of Internal Medicine Leiden University Medical Center Leiden Netherlands; 3 Department of Public Health and Primary Care Leiden University Medical Center Leiden Netherlands; 4 National eHealth Living Lab Leiden Netherlands; 5 Department of Design, Organization and Strategy Faculty of Industrial Design Engineering Delft University of Technology Delft Netherlands

**Keywords:** diabetes mellitus, type 2, telemedicine, self-management, patient education as topic, activation, glucose regulation, Center for eHealth and Wellbeing Research, CeHRes, type 2 diabetes, tele, patient education, CeHRes roadmap, diabetes, glucose, insulin, education, development, usability, medical, behavioral, psychological, digital consultation, feasibility, endocrinology, endocrine, focus group, dietitian, psychologist, nurse, lifestyle factor, diet, exercise, stress, sleep, cardiovascular disease, heath care professional, mobile phone

## Abstract

**Background:**

Type 2 diabetes (T2D) tremendously affects patient health and health care globally. Changing lifestyle behaviors can help curb the burden of T2D. However, health behavior change is a complex interplay of medical, behavioral, and psychological factors. Personalized lifestyle advice and promotion of self-management can help patients change their health behavior and improve glucose regulation. Digital tools are effective in areas of self-management and have great potential to support patient self-management due to low costs, 24/7 availability, and the option of dynamic automated feedback. To develop successful eHealth solutions, it is important to include stakeholders throughout the development and use a structured approach to guide the development team in planning, coordinating, and executing the development process.

**Objective:**

The aim of this study is to develop an integrated, eHealth-supported, educational care pathway for patients with T2D.

**Methods:**

The educational care pathway was developed using the first 3 phases of the Center for eHealth and Wellbeing Research roadmap: the contextual inquiry, the value specification, and the design phase. Following this roadmap, we used a scoping review about diabetes self-management education and eHealth, past experiences of eHealth practices in our hospital, focus groups with health care professionals (HCPs), and a patient panel to develop a prototype of an educational care pathway. This care pathway is called the Diabetes Box (Leiden University Medical Center) and consists of personalized education, digital educational material, self-measurements of glucose, blood pressure, activity, and sleep, and a smartphone app to bring it all together.

**Results:**

The scoping review highlights the importance of self-management education and the potential of telemonitoring and mobile apps for blood glucose regulation in patients with T2D. Focus groups with HCPs revealed the importance of including all relevant lifestyle factors, using a tailored approach, and using digital consultations. The contextual inquiry led to a set of values that stakeholders found important to include in the educational care pathway. All values were specified in biweekly meetings with key stakeholders, and a prototype was designed. This prototype was evaluated in a patient panel that revealed an overall positive impression of the care pathway but stressed that the number of apps should be restricted to one, that there should be no delay in glucose value visualization, and that insulin use should be incorporated into the app. Both patients and HCPs stressed the importance of direct automated feedback in the Diabetes Box.

**Conclusions:**

After developing the Diabetes Box prototype using the Center for eHealth and Wellbeing Research roadmap, all stakeholders believe that the concept of the Diabetes Box is useful and feasible and that direct automated feedback and education on stress and sleep are essential. A pilot study is planned to assess feasibility, acceptability, and usefulness in more detail.

## Introduction

### Background

Around 1 in 11 adults in Europe have diabetes mellitus, and the number of people with diabetes is increasing [1]. Currently, in more than 95% (n>1 million) of diabetes cases, it concerns type 2 diabetes (T2D). In people who are genetically predisposed to diabetes, adverse eating habits, excess body weight, and physical inactivity induce disruption of glucose control [2]. Hence, healthy lifestyle behaviors play a critical role in preventing and managing T2D. Indeed, quitting smoking, being more physically active, eating healthier, and losing weight when overweight can significantly reduce the risk of developing T2D [3,4]. Furthermore, in patients with recently diagnosed T2D, it was demonstrated that dietary lifestyle interventions can lead to persistent diabetes remission after 24 months in 36% (n=149) of patients [5].

Despite the obvious benefits of healthier lifestyles, adherence to healthy lifestyle behaviors in patients with T2D is poor [6-8]. This is alarming, as worse adherence obviously hampers therapeutic efficacy [9]. In Europe, glucose control is inadequate in at least half of the people with T2D. Inadequate glycemic regulation increases the risk of diabetes-related complications and mortality, and it increases medication use and health care costs [10,11]. Immediate action is needed to halt the rising incidence of T2D as well as to decrease the burden of T2D and curb health care costs [1].

A Cochrane review showed that diabetes self-management education (DSME) in people with T2D can improve glucose regulation [12]. In addition, it potentially improves blood pressure and reduces body weight and the requirement for diabetes medication. Yet, another systematic review reported that encouraging patients to play an active role in self-management, so-called patient activation or empowerment, can also improve glucose regulation [13]. Notably, mounting evidence clearly shows that the physiological response to lifestyle change is highly personal [14,15]. Moreover, it seems obvious to suppose that home monitoring of medical and behavioral parameters stimulates and improves self-management. Indeed, integrative monitoring of lifestyle behaviors and physiology using direct action-feedback loops potentially allows for the provision of informative personalized lifestyle advice [16].

Traditionally, DSME is done face-to-face, but digital tools can facilitate health behavior change and significantly improve glucose regulation in patients with T2D [17]. Effective digital tools are self-monitoring (eg, continuous glucose monitoring [CGM]) and telemonitoring by health care professionals (HCPs) [18,19]. Mobile phone apps providing automated feedback can also be effective in improving lifestyle modification and glucose regulation for people with T2D [20]. Indeed, due to low costs as compared to health care consultations and the 24/7 availability of HCPs, mobile phone apps have a lot of potential in diabetes management [21]. Currently available eHealth tools usually focus on one particular lifestyle component or relevant clinical parameter, such as CGM devices or apps that facilitate counting carbohydrates. Examples of digital tools that combine different lifestyle and biometrical parameters to improve self-management and glycemic control exist [22-24]. However, only a few digital tools exist that combine behavioral as well as biological data to provide informed, personalized lifestyle advice to people with T2D [24]. Most of the existing tools are one-size-fits-all lifestyle solutions. Personalized interventions are preferred as clinicians and patients together can choose the treatment plan that contributes most to favorable patient outcomes [25]. Here, we aimed to develop an eHealth-supported educational pathway using integrated behavioral and biological data collected by home monitoring to provide personalized lifestyle advice and promote self-management of people with T2D. Early involvement of stakeholders in the development process of eHealth tools is paramount for successful implementation in health care [26-30]. To assist in the construction of successful eHealth technologies, the Center for eHealth and Wellbeing Research (CeHReS) designed a roadmap to guide eHealth device development, implementation, and evaluation. The CeHReS roadmap consists of 5 phases and emphasizes stakeholder involvement throughout all of these phases [31]. The CeHReS roadmap was used to construct our educational program.

### Objectives

Our aim is to empower patients with T2D to manage their disease by developing an integrated, eHealth-supported, blended educational pathway called the Diabetes Box (Leiden University Medical Center). In this paper, we delineate the different phases of the participatory development of the Diabetes Box using the CeHReS roadmap, and the lessons learned are shared.

## Methods

### Ethical Considerations

The accredited medical research ethics committee Leiden den Haag Delft (MREC registration P21.045) has reviewed the research protocol and gave its approval. The patients participating in the panel provided informed consent for their feedback and input to be used in scientific publication. Input data were deidentified. No compensation was provided for participating in the panel.

### CeHReS Roadmap

**Figure 1:**
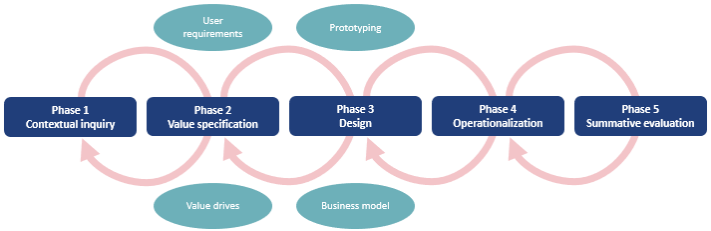
Overview of the CeHRes roadmap showing the different phases and formative evaluation (adapted from van Gemert-Pijnen et al [31]). CeHReS: Center for eHealth and Wellbeing Research.

The CeHReS roadmap was used to guide the development process of the Diabetes Box [31]. The CeHReS roadmap was designed to assist in planning, coordinating, and executing the development process of eHealth tools. The roadmap has a participatory dynamic and consists of 5 intertwined phases and continuous formative evaluation (Figure 1). The first 4 phases (ie, contextual inquiry, value specification, design, and operationalization) of the development process of the Diabetes Box are presented in this paper. The summative evaluation will be performed when the Diabetes Box is launched.

The contextual inquiry is meant to understand the challenges faced by the main stakeholders and how they could be solved. To this end, a literature review was performed. We followed the stages of a scoping review according to the revised Arksey and O’Malley framework [32]. We specified the research question “What diabetes self-management education strategies are being used in regular medical care?” The search strategies combined the terms “diabetes self management education,” “technology/telemonitoring/glucose monitoring,” and “healthcare/medical care.” We searched PubMed and used Google for a broader search. One researcher (DLF) selected the studies and discussed these with a team consisting of 2 endocrinologists, a psychologist, a dietitian, and 2 diabetes nurses, all experienced in the field of DSME. Relevant studies were selected and summarized after which the team discussed the report. To elaborate further on the review of literature, previous experiences with eHealth in our center were evaluated, and important stakeholders were identified and interviewed in focus groups. Previous experiences mainly included technological and practical considerations from implementations of eHealth for patients with myocardial infarction, cardiac surgery, and COVID-19 [33-35]. The main stakeholders were patients, medical specialists, dietitians, psychologists, and diabetes nurses. The latter 4 would later form the development team and partake in the first 2 focus groups.

During the second phase, the value specification, the values gathered in the first phase were translated into (technological) requirements. What problems should the tool solve and how should it work? Weekly meetings with the relevant stakeholders (identified during the contextual inquiry) were used to refine the values and specify the technological requirements of the Diabetes Box.

Using these requirements, prototypes of the Diabetes Box were created during a highly dynamic, iterative, and collaborative design phase. Through biweekly meetings, the development team and stakeholders collaborated closely to ideate, create, and discuss ideas. A panel of patients with T2D gave feedback on the prototype. The entire development team was present on the web during the patient panel. Two members of the team wrote a summary of the recording, after which the recording was deleted. The entire team came together to discuss the outcomes of the patient panel, extract the most important aspects, and set out to change the prototype accordingly. Throughout the development process, the development team looked back on values and knowledge from previous phases to check the integrity of the design. Furthermore, at any point, incoming information could lead to adaptions in the process. This formative evaluation was enabled by constantly involving stakeholders in evaluations and decision-making.

When the design satisfied all stakeholders, the operationalization phase began. During this phase, the Diabetes Box was put into practice. First, a plan was made to implement the newly developed technology into the context defined by the contextual inquiry. The plan was made in close cooperation with the stakeholders to ensure a good fit. Second, the technology is launched.

**Figure 2:**
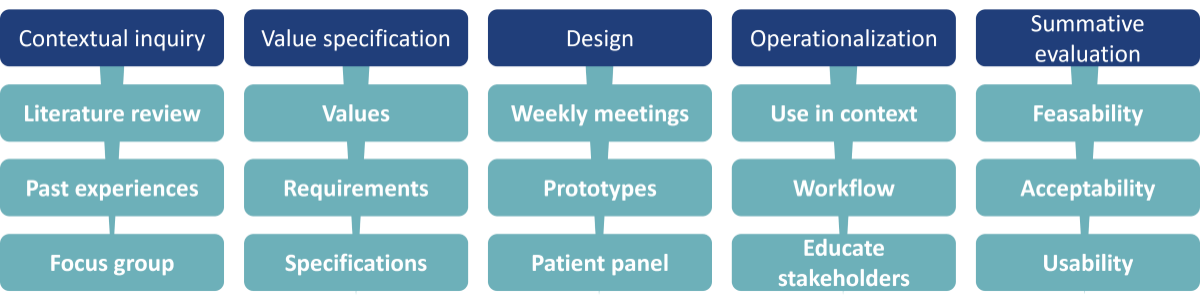
Overview of aspects in every phase. Note that this paper focuses on the contextual inquiry, the value specification, and the design phase [31].

In the fifth and last phase, the summative evaluation, the tool will be tested in the real world. Currently, the development team is setting up a pilot study to evaluate the feasibility, acceptability, and usability in clinical practice and get an impression of the clinical effects. It is important to note that the technology is quite versatile and adaptable to suit the practical demands of stakeholders as revealed during phase 5. A summary overview of all phases in this study is presented in Figure 2.

## Results

### Phase 1: Contextual Inquiry

The contextual inquiry was meant to understand the challenges faced by the main stakeholders and how these challenges could be solved. We used a review of the literature and evaluated past experiences and focus groups with important stakeholders.

#### Literature Review

The information gathered from the literature review is summarized in Table 1. Studies have outlined several ways to support self-management, which can be categorized as education, monitoring, and modalities. Evidence shows that group-based education about disease pathophysiology, the influence of lifestyle (diet, exercise, stress, and sleep), self-management, and patient activation can improve glucose regulation, reduce body weight, and reduce the need for diabetes medication [4,12,17]. However, HCPs generally feel that they are insufficiently equipped to provide patients with T2D with the insights required to facilitate their health-related behavior change [36-38]. Furthermore, studies suggest that dietician-led lifestyle intervention as compared to interventions led by other HCPs achieves greater weight reductions [39].

Studies on monitoring indicate that self-monitoring (patients monitoring their own health parameters) and telemonitoring (using information technology to monitor patients at a distance) can significantly increase glucose regulation and reduce T2D-related complications [19]. For example, CGM significantly improves glucose regulation and reinforces patient satisfaction [18,48]. In addition, even though activity tracking has ambiguous effects on glucose regulation, it appears to reduce mortality and CVD risk in patients with T2D as well as the incidence of T2D in a general population [51,52]. Furthermore, blood pressure monitoring can decrease systolic blood pressure in patients with T2D when supported by an HCP [53]. To our knowledge, weight monitoring has not been assessed as a stand-alone intervention, but focus on weight can lead to stigma in patients with T2D, potentially leading to increased emotional distress [55].

**Table 1:** Outcomes of literature review.

Effects			Comments
**DSME^a^**			
	Self-management ↑^b^ Glucose regulation ↑ [4,12,13,39] Knowledge or insight ↑ [12] Body weight ↓^c^ [12,39] Need for medication ↓ [12] Blood pressure ↓ [12]	The inclusion of disease pathophysiology contributes to the effect of DSME [12] Discussing the influence of lifestyle factors (diet, exercise, sleep, and stress) in DSME is decisive for its improvements [4,12,40-46] Empowerment and patient activation beyond mere education are important in DSME [13] Digital components of education can be effective [17,39] The involvement of a dietitian increases the effect on body weight [39]	
**Telemonitoring**			
	Glucose regulation ↑ [19] Diabetes-related complications ↓ [19]	Manual input may lead to erroneous input and can lower compliance [47]	
**CGM^d^**			
	Glucose regulation ↑ [18,48] Patient satisfaction ↑ [49]	Failing to integrate well-structured education in glucose monitoring can diminish the effects on glucose regulation [50]	
**Activity tracker**			
	Glucose regulation ↑/↓^e^ [51] Incidence T2D^f^ ↓ [52] Mortality ↓ [51] CVD^g^ risk ↓ [51]	N/A^h^	
**Blood pressure monitor**			
	Systolic blood pressure ↓ [53]	HCP^i^ support increases the effect [53,54]	
**Weight monitoring**			
	Not assessed as a stand-alone	Focus on weight monitoring and loss can be stigmatizing and lead to increased diabetes-related distress [55]	
**Mobile apps**			
	Glucose regulation ↑ [56] Monitoring or education ↑ Lifestyle modification ↑ [20]	HCP support increases the effect [56-58] User-friendliness is an important aspect for success [58] Apps can provide insight into self-management [57]	
**Dietary journal**			
	Inform patients ↑ Evaluate interventions ↑	An easier, less time-consuming method would be beneficial to adherence Photos have equal results as food weighing [59]	

^a^DSME: diabetes self-management education.

^b^↑: improves.

^c^↓: deteriorates.

^d^CGM: continuous glucose monitoring.

^e^↑/↓: ambiguous results.

^f^T2D: type 2 diabetes.

^g^CVD: cardiovascular disease.

^h^N/A: not applicable.

^i^HCP: health care professional.

As far as modalities are concerned, mobile phone apps have a lot of potential in T2D management due to low costs, 24/7 availability, and dynamic automated feedback [21]. Evidence points out that mobile phone apps providing lifestyle advice can improve glucose regulation and facilitate lifestyle modification, particularly when they are supported by high-frequency HCP feedback [20,56,57]. Furthermore, keeping electronic dietary records effectively informs patients about the impact of food on glucose levels, but easy-to-use technology is needed [60,61]. For example, taking pictures of meals may be an adequate alternative of time-consuming, labor-intensive recording of dietary components [59].

#### What Experiences do we Have With Digital Tools Supporting Self-Management?

##### The Box

The Box (Leiden University Medical Center) comprises a set of eHealth tools that aim to improve self-management skills for a specific chronic condition. It includes devices for home monitoring of biological and behavioral parameters relevant to health (eg, glucose concentrations, physical activity, or blood pressure). The data are presented to the patient in a smartphone app called the LUMCCare app (discussed in LUMCCare App section). The data are also sent to the patients’ electronic medical records in the hospital to allow evaluation by HCPs. The efficacy and safety of the Box have been examined in the follow-up care of patients with myocardial infarction. We recently reported that patient satisfaction with the Box was equal to regular medical care and that 96% (n=100) of participants appreciated that they could view their health data [62]. Furthermore, the Box has been shown to reduce hospital admissions by effectively surveying clinical symptoms and vital signs at home in patients with COVID-19 [35]. In conclusion, the Box appears to be an effective and appreciated prototype instrument for home monitoring that can be tailored to the health care needs of different conditions.

##### LUMCCare App

**Figure 3:**
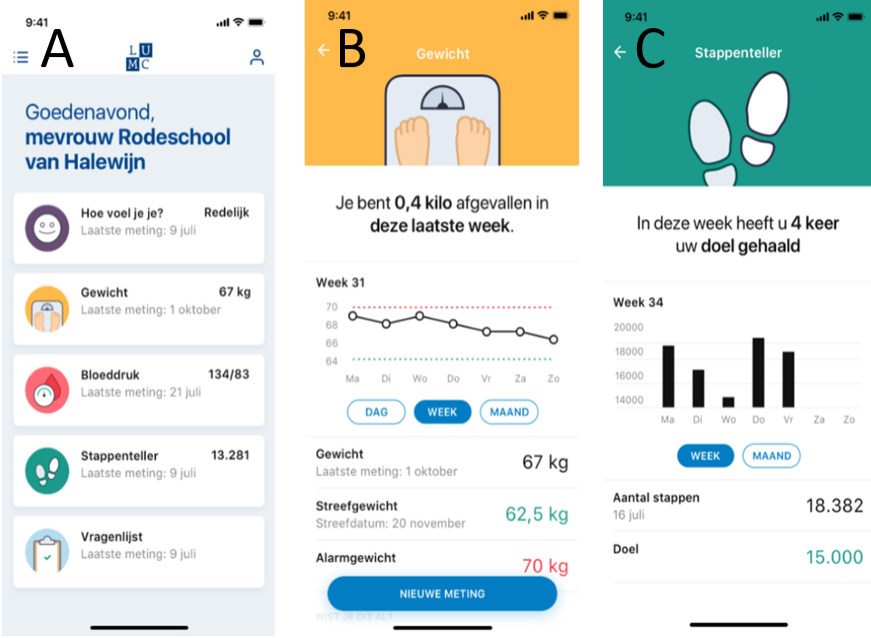
Screenshots of the LUMCCare app from left to right: (A) the home screen showing general well-being, weight, blood pressure, activity, and questionnaires, (B) the weight screen, and (C) the activity screen.

The LUMCCare app (Leiden University Medical Center) is a smartphone app available for Android and iOS. All data collected by the devices in the Box are automatically sent to the LUMCCare app via Bluetooth. The LUMCCare app was codeveloped with people with low health literacy, ensuring a good understanding and usability also in those individuals. Currently, the app was developed in the Dutch language and can display measurements of weight, blood pressure, heart rate, electrocardiograms, steps, temperature, and oxygen saturation (Figure 3). Moreover, users can indicate their level of well-being and provide a brief explanation. HCPs can also send questionnaires to patients through the app. The vast majority of patients with myocardial infarction report intensive and consistent use and high satisfaction with the app [62].

#### Stakeholders, Current Situation, and Experiences

The main stakeholders included patients, medical specialists, dietitians, psychologists, and diabetes nurses. To evaluate and confirm the findings of our scoping review and past experiences, we organized focus groups with a professor of diabetology, a clinical endocrinologist, a dietician, a psychologist, 2 diabetes nurses, an IT specialist, and a researcher.

According to international guidelines, people with T2D at least annually visit a physician (endocrinologist or general practitioner) and a nurse specialized in diabetes care. These HCPs should educate patients on (the role of lifestyle in) the pathophysiology of diabetes and on the types and dosing of available medication. Based on patient needs and health parameters, they decide if the patient requires a consult with the dietician or psychologist. All international guidelines advocate lifestyle intervention as a first step in the treatment of T2D. However, health care systems generally lack the means to adequately support patients trying to change deeply engrained habits. This is made exceedingly difficulty by an environment that relentlessly entices them to make unhealthy choices. All HCPs confirmed that continuous home monitoring of subcutaneous glucose concentrations has been a significant advance in supporting and motivating patients with diabetes to enhance their own grip on disease management. The notion that home monitoring of various relevant behavioral and biological parameters and integrating the data to yield personalized feedback would enhance patient empowerment and potentially improve self-management was broadly shared. To these ends, the contents of the Box and LUMCCare app were envisioned to require specific features as further defined in the next stage of development. A summary of the current situation is provided in Textbox 1.

**Current situation of health care for patients with type 2 diabetes (T2D) according to health care professionals (HCPs).**

**What is going well?**

- Knowledge on diet, exercise, stress, and sleep is intermittently conveyed to patients by HCPs.
- All patients see an endocrinologist and diabetes nurse. If deemed necessary, a dietician and a psychologist are available.
- Adequate optimization of medication use.
- Close interdisciplinary collaboration between doctors, dieticians, psychologists, and diabetes nurses in the care for patients with diabetes.
- Health care can be delivered through digital means.

**What can be improved?**

- Patients’ knowledge regarding the influence of lifestyle behaviors (diet, exercise, sleep, and stress) on glucose regulation.
- Activation of patients with T2D to improve lifestyle behaviors.
- Personalized lifestyle advice.
- Focus on personal goal setting.
- Shared decision-making regarding the timing and intensity of consultations with HCPs.
- Home monitoring of relevant parameters.
- Digital group consultations.

### Phase 2: Value Specification

After a thorough exploration of the context and potential improvements, the next step was to translate the requirements of eHealth tools that were identified by the HCPs into specific technological properties. First, HCPs emphasized the need to more extensively convey the importance of lifestyle behaviors, including diet, exercise, stress, and sleep, in the control of glucose metabolism and the treatment of T2D. HCPs also stressed that the tool should tailor information and advice to the needs and wishes of the patient and that it should be easy to use for both patients and HCPs. Moreover, it should have features that activate patients to appropriately adapt their lifestyle. Activation was listed as a separate capacity of the eHealth tool. Finally, and importantly, the capability to monitor relevant parameters at home and easy accessibility to collected data for patients and HCPs were defined as prerequisites of an effective tool. This leads to a complete list of values, tool requirements, and tool specifications (Table 2).

**Table 2:** User perspective, user values, tool requirements, and tool specifications of the Diabetes Box.

Values	Tool requirements	Tool specifications
Provide insight, holistic view	Provide tailored education on the relationship between specific lifestyle factors and glucose regulation	Include a graph of glucose combined with relevant lifestyle factors (diet, activity, sleep, and stress). Include education on these topics.
Activate and stimulate	Help stimulate patients to adopt healthy behaviors	Include goal setting in all aspects of self-management education. Provide direct behavior–related feedback and education.
Personalized	Tailored to the patient	Provide a place where patients can monitor their own personal and combined parameters.
24/7 Availability	Rely mostly on apps and e-learnings that are available 24/7	Provide a digital resource that patients can use in their own time to measure glucose, diet, activity, stress, and sleep. Provide links for access. Resources or videos.
Integrated in health care	Integrated in health care	Add Diabetes Box dashboard to the electronic medical record. Plan education by HCPs^a^ in work hours.
User-friendly	Easy to use, logical, and understandable	Use B1-level language throughout the tool. Simplify user interface. Incorporate dashboard into electronic medical record. Align education contents with the expertise of HCPs.
Monitoring patients	Monitoring of patient parameters and making them available for patient and HCP	Provide a place where patients can gain insight into personal parameters of lifestyle factors and glucose. Provide a dashboard where HCPs can monitor combined patient parameters to provide tailored support.
Low costs	No extra costs for patients, lower costs for health care	Build on existing app content. Use group meetings. Free for patients (insurance covered).
Service desk	Enable a patient support desk	Two separate phone numbers were provided for difficulties. First, the outpatient clinic number for diabetes-related questions, and second, the Box support desk for technology-related questions.

^a^HCP: health care professional.

### Phase 3: Design

#### Overview

**Figure 4:**
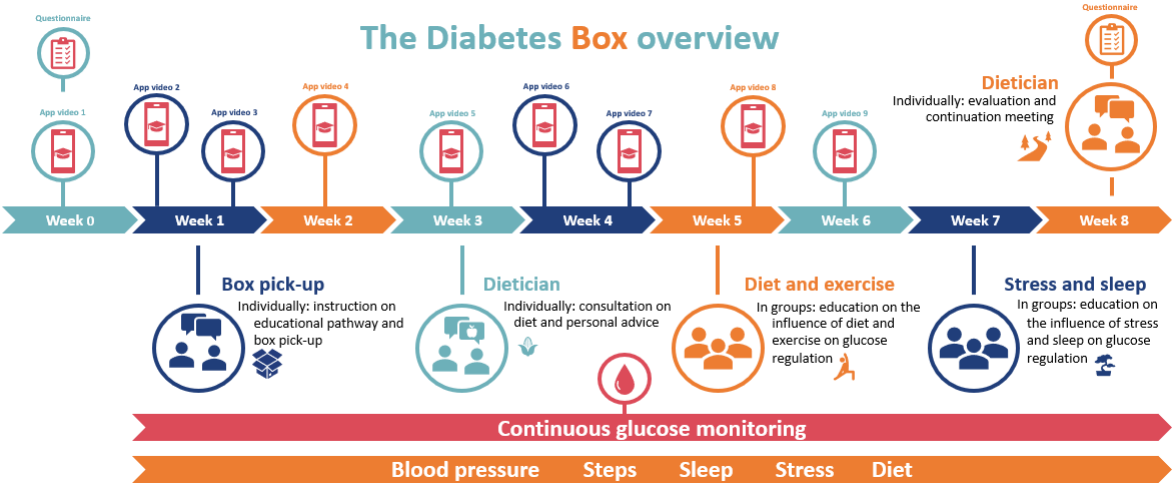
Overview of the educational pathway for patients with T2D using the Diabetes Box.

**Figure 5:**
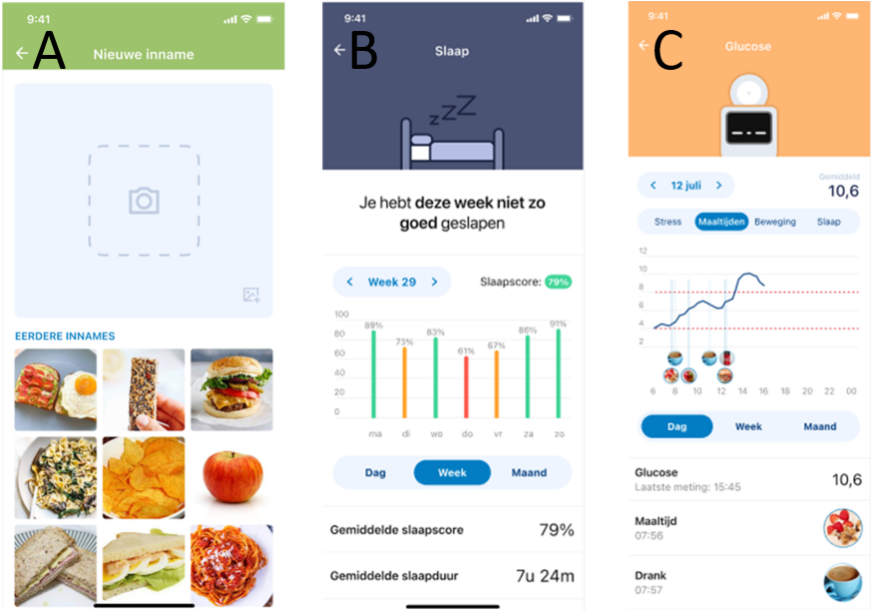
Screenshots of the new modalities of the LUMCCare app from left to right: (A) the screen to register a new intake, either food or drink, with a photo; (B) the sleep screen showing an average score based on estimated duration, interruptions, and regularity; and (C) the glucose screen combined with the diet screen showing the photos of intakes in the glucose graph.

The Diabetes Box was developed using the participatory development method guided by the CeHReS roadmap. The design revolves around making prototypes of the Diabetes Box based on the tool specifications identified in the previous phases and gathering feedback from stakeholders. Our initial prototype was presented to HCPs and shared in a patient panel described below. After feedback from the HCPs, the Diabetes Box comprised digital self-measurement tools, an app, and DSME in the form of consultations and instructive videos (Figure 4). The tools included a continuous glucose monitor (Abbott Freestyle Libre), a sleep or activity tracker (Withings HR Steel), and a blood pressure monitor (Withings BPM Connect). The data collected were presented in the LUMCCare app. Subjective stress could also be registered, and food intake could be monitored by pictures taken of all that was consumed (Figure 5). All data were easily visualized in daily, weekly, and monthly overviews. The data of diet, activity, sleep, and subjective stress could also be plotted on the continuous glucose graph to provide insight into the relation between lifestyle factors and glucose regulation. An expert-led educational program was developed to further promote knowledge of the relationships between various components of lifestyle and glucose control. The overarching goal of the Diabetes Box is to empower patients and facilitate self-management of their disease. The program combines knowledge from routine diabetes care provided by dietitians, psychologists, endocrinologists, and specialized nurses. All educational material was developed, aiming to promote patient self-management. Therefore, multiple behavior change techniques were included in the development of the Diabetes Box. These included information provision, goal setting, action planning, self-monitoring, feedback provision, social comparison, and motivational interviewing. In Multimedia Appendix 1, we provide a list of behavior change techniques as described by Michie et al [63], including a description of the app in the Diabetes Box. There were nine 3- to 5-minute educational videos combining live feed and animations. The topics of these videos were an introduction to the Diabetes Box, the pathophysiology of diabetes, CGM, diet, exercise, sleep, stress, goal setting, and self-management. The educational program also entailed five consultations: (1) a group consultation introducing the digital tools, (2) an individual consultation focusing on diet, (3) a group consultation regarding diet and exercise, (4) a group consultation regarding sleep and stress, and (5) an individual consultation to evaluate, conclude, and set up future goals. Goal setting and patient activation were present in all videos and all educational consultations. The consultations lasted 45-90 minutes. Prior to each consultation, participants were asked to watch 1 or 2 videos.

#### Patient Panel

The prototype of the Box was shared with people with T2D to gather feedback on the preliminary design. Due to COVID-19 measures, only 4 people with diabetes were present during the session. The session took 90 minutes. The concept of the Box program was explained, 2 of the 9 educational videos were shown, the app and its functionalities were demonstrated, and people could try out the eHealth tools. The panel was asked questions covering 4 domains: general opinion, contents and clarity of the educational videos, functionality of eHealth instruments, and usability of the app. Overall, the evaluation was positive, while several potential improvements were suggested (Table 3).

Based on the feedback provided by the patient panel, multiple adaptations were made. First, a leaflet was added to the Box, explaining the flow of the program and anticipated time investment from the side of patients in more detail. Second, a web page was made, displaying the videos accompanied by instructions on when to watch which video. Third, a handout was made with detailing information about the different types of diabetes medications, their uses, and their common side effects. Fourth, as patients preferred a mix of consultation types, 2 consultations were planned online and 3 face-to-face. Fifth, the video of sleep was cut into 2 halves of 3 minutes to prevent viewers from quitting halfway through. Last, the LUMCCare was further developed to also accommodate insulin registration and other activities than steps (eg, cycling or swimming).

**Table 3:** Outcomes of patient panel.

Comments			Actions
**General opinion**			
	Positive first impression Useful and feasible Questions about total time investment, duration of the education, and loan of the devices Advise to restrict the number of needed apps to one	Informative material was made for participants of the Diabetes Box addressing the expected time investment, duration of the pathway, and the fact that participants can keep the devices. Technologically we still need 2 apps. However, 1 only needs to run on the background and does not have to be opened.	
**Education**			
	Positive Duration and frequency seem feasible Half preferred online (travel distance and comfort of home) and half preferred live (connection with HCP^a^) Early evening is the best time Videos are appreciated (up to 3 minutes) A clear overview of videos and when to watch them is needed Advice in education has to be consistent Extra attention for diabetes medication, not all glucose levels can be related to behavior, correct use of the CGM^b^	Hybrid pathway, part of consultations live, part online. The video of sleep was cut into 2 parts. A web page was made with an overview of all videos explaining when to watch which. An extra handout was made about diabetes medication. Attention to explaining glucose levels was added to the information.	
**Devices or measurements**			
	Doable, clear, and easy to use Frequency of measurement was regarded positively Activities other than steps would be great A (3 hours) delay in showing glucose values was deemed very impractical	Added activity tracking other than steps in the app. Focused on displaying real-time glucose data in the app.	
**LUMCCare app**			
	Positive about layout and readability Stress measurement and diet photos were deemed useful Diet photos were deemed confronting in a helpful way Incorporate insulin use in the app	A functionality to register insulin use was added to the LUMCCare app.	

^a^HCP: health care professional.

^b^CGM: continuous glucose monitoring.

### Phases 4 and 5: Operationalization and Summative Evaluation

Operationalization involves the introduction of eHealth technology into practice. To test our design, we are currently planning a pilot study in 32 people with T2D to assess the feasibility, acceptability, and usability of the Box in clinical practice. Secondary objectives are evaluation of time in range and perceived learning. The study duration will be 2 months (as the concept Box program lasts 2 months). Participants will fill out a questionnaire before and after the study, and they will be interviewed about their experience as well. Patient satisfaction, user-friendliness of Box components, added value of the program in terms of disease management, and eventual use of the help desk will be evaluated. Consultation attendance, the use of eHealth tools and apps, and eventual replacement of glucose monitors will be registered. HCPs will be asked for their opinion regarding clinical practicalities in a structured interview, and average health care costs will be calculated.

## Discussion

### Principal Findings

Personalized lifestyle advice and promotion of self-management can help patients change their health behavior and improve glucose regulation. Digital tools have great potential in supporting patient self-management due to the effectiveness, low costs, 24/7 availability, and the option of dynamic automated feedback. However, reports documenting the impact of interventions incorporating multiple lifestyle modalities on glucose control are, to our knowledge, not available. Here, we developed an integrated, eHealth-supported, educational care pathway for people with T2D following the CeHReS roadmap and using a scoping review about DSME and eHealth, past experiences of eHealth practices in our hospital, focus groups with HCPs, and a patient panel. The care pathway aims to empower patients with T2D to self-manage their disease by providing them with direct feedback on their personal health behavior in relation to contemporaneous glucose levels.

HCPs and patients thought the concept of the Diabetes Box to be feasible, acceptable, and useful. The main strengths of the Diabetes Box were considered to be the integration of direct biofeedback on personal behavior, the focus on goal setting, and patient activation.

### Comparison to Prior Work

The direct biofeedback regarding the impact of behavior on glucose concentrations was believed to be crucial to provide patients with insight into the relationships between their health behavior and glycemic control. A similar conclusion was drawn in an earlier study where patients with T2D were motivated to exercise while using CGM and accelerometer technology [64]. In many studies, data on lifestyle parameters were entered manually or via voice recording [22,24]. A Korean study showed input rates of diet and exercise of 24.9% and 5.3%, respectively [22]. Our study uses automatic input of steps, sleep, blood pressure, and glucose levels facilitating data gathering by participants. Diet was tracked using photographs. These photos were not analyzed for caloric content or carbohydrates but were used to provide insight into glucose-level fluctuations caused by certain food types. Beyond automated recording of behavioral and biological parameters, our app enables combining all lifestyle parameters with continuous glucose levels to create easily interpretable relations between lifestyle and glucose levels.

Regarding these lifestyle components, other interventions for people with T2D focus primarily on diet and exercise [23,24,65]. In one German study, stress management was included in the educational material, but stress or mood was not measured during the study [66]. The lifestyle components on which feedback should be provided include diet, physical activity, stress management, and sleep. Chronic stress may be a less obvious yet important disruptor of glucose control, as indicated by previous research [67,68]. Indeed, a recent meta-analysis revealed that stress reduction therapy improves glycemic control in people with diabetes [46]. The Diabetes Box gives direct biofeedback on diet, exercise, sleep, and stress. The effect of direct biofeedback on personal behavior is further enhanced by structured and tailored education. This is important, as physiological responses to lifestyle changes are often determined by personal characteristics [14,15]. Other studies often use one-size-fits-all education or even automated SMS text messages [24,65,69]. During the educational consultations in the Diabetes Box, HCPs can inform patients regarding the effects of their personal health behavior on metabolic control. The education in the Diabetes Box was designed to be simple, patient-centered, and multimodal, which is in line with the literature on successful patient education [70,71].

In addition, most of the existing tools are used in a research setting, and the challenge is to integrate these tools into regular medical care. A recent Dutch study showed that following a 2-year multicomponent lifestyle program outside of regular medical care could reduce medication use. In this setting, 71% of insulin users could stop insulin, and 28% of participants could stop glucose-lowering medication altogether. It must be stated though that only 234 of 438 starting participants were used in the final analyses [23]. When using these tools as regular medical care, all patients with T2D will follow the program instead of a selection of the more motivated patients. Our setup is to use this tool as regular medical care for all patients with T2D. The participatory development with an entire endocrinology team working in diabetes care can improve the chance of successful implementation. We are curious if similar results will be achieved when a multicomponent lifestyle program is integrated into regular medical care.

Studies have shown that people with higher levels of lifestyle-related knowledge (eg, influence of diet on glucose levels) tend to make healthier choices to improve their glycemic control [72]. However, better education and insight do not necessarily translate into behavior change [73]. To stimulate patients to change their behavior, goal setting and activation are integrated into all components of the Diabetes Box. All videos end with an assignment to self-monitor specific behavioral and biological parameters in preparation of the next consultation, and a separate video about goal setting is included. Furthermore, the individual consultations with the dietitian revolve around diet but also cover reflection on goals set by a patient. It is difficult to empower people with insufficient diabetes-related knowledge to manage their disease [74]. Therefore, we believe it is the combination of direct feedback, structured and tailored education, and targeted patient activation that grants the Diabetes Box its great potential.

The participatory development process played a critical role in the realization of the Diabetes Box. Involving all stakeholders from the start proved very fruitful, as it clearly facilitated the creation of a program that fits all stakeholder demands. The CeHReS roadmap was very helpful as well. It provided handholds and courses of action, which make it easier to make and measure progress. In addition, the value specification generated a concrete set of wishes from the key stakeholders that could be used to fall back and make decisions.

### Limitations and Strengths

Obviously, there are at least 2 issues that limit the broad-based application of the Diabetes Box for the time being. First of all, the feasibility of the program as well as its impact on metabolic control and quality of life of patients with T2D needs to be evaluated in clinical practice. As the program was primarily developed by stakeholders employed by a third-line, academic medical institution, it also needs to be tested if it works for patients under regular surveillance by primary care (n>1 million, 90% of patients with T2D are treated by their general practitioners in the Netherlands). Second, although the LUMCCare app was created as a “white label” app, which means that it is relatively easy to adapt external characteristics, it was designed as part of the local (Leiden University Medical Center) infrastructure. Use by other institutions would therefore probably require modifications. We are willing to help and assist hospitals and other health care institutions that want to implement the Diabetes Box into their regular medical care for people with T2D. The challenges we foresee are training personnel and integrating the Diabetes Box into their daily workflow. The type of specialist who provides the consultations can be changed depending on what professionals are motivated and at hand. In addition, the content of the educational material can be altered to better fit the personal approach of the professional providing the education. On a technological basis, challenges also exist. The app is white label and can be easily adapted to accommodate the look and feel of other institutions. However, the data generated in the app have to be made available for the HCPs involved. This will most commonly involve integration into the electronic medical records, which is a process that costs both time and money. In the near future, the Diabetes Box will be tested in a single-center, mixed methods, sequential explanatory pilot study including approximately 32 patients with T2D, with the primary aim to assess its feasibility, acceptability, and usability. Secondary objectives will be to evaluate its impact on the “time in range” of glucose levels and perceived learning. Subsequently, in case of promising results, the Diabetes Box will be tested for efficacy in a larger, multicenter (including primary care) intervention study.

### Conclusions

We have developed a unique care pathway in close collaboration with relevant stakeholders in order to ensure a good fit. The combined effects of direct biofeedback on personal behavior, structured and tailored education, and goal setting should empower people with T2D to improve their self-management and glycemic control. A pilot study is planned to assess feasibility, acceptability, and usability in more detail.

## Appendix

Multimedia Appendix 1 Description of behavior change techniques used in the Diabetes Box.

## Abbreviations

CeHReS: Center for eHealth and Wellbeing Research
CGM: continuous glucose monitoring
DSME: diabetes self-management education
HCP: health care professional
T2D: type 2 diabetes
