# Supplementary material for: Participatory Development of an Integrated, eHealth-Supported, Educational Care Pathway (Diabetes Box) for People With Type 2 Diabetes: Development and Usability Study
Source: JMIR Hum Factors. 2024 May 31;11:e45055. doi: 10.2196/45055 (PMC11179029; doi:10.2196/45055)
Supplement: Multimedia Appendix 1 [file humanfactors_v11i1e45055_app1.docx]

| Behavior change techniques used in the Diabetes Box | Description |
| --- | --- |
| Provide information on consequences of lifestyle behavior on *glucose levels in general* | General education on diabetes pathology  Clear outlining of influence of lifestyle factors (diet, exercise, sleep and stress) on glucose levels |
| Provide information on consequences of lifestyle behavior for *the individual* | Explanation that healthier lifestyle behavior can lead to better regulated glucose levels, fewer complications and fewer medications |
| Goal setting (behavior) | Goals should be specific, measurable, acceptable, realistic and time-bound (SMART)  Participants are encouraged to set goal(s) on lifestyle behavior |
| Goal setting (outcome) | Secondary to behavior, blood glucose regulation and blood pressure reduction are used as goals |
| Action planning | When a participant chooses a certain goal, action plans are made |
| Provide rewards contingent on healthy behavior | Compliments are granted upon changing health behavior  Special attention is given to improved glucose levels after behavior change |
| Prompt self-monitoring of lifestyle behavior | Behavior is recorded in the LUMCCare App  Participants are encouraged to evaluate health behavior using the app |
| Prompt self-monitoring of lifestyle behavioral outcome | Glucose levels and blood pressure are presented in the app  Encouragement to monitor blood glucose in combination with health behavior |
| Provide feedback on performance | Immediate fluctuations in glucose levels can be linked to health behavior |
| Provide instruction on how to perform healthy lifestyle behavior | Instruction on diet, exercise, stress and sleep, during educational sessions and individual consultations  The App provides tips on these lifestyle behaviors |
| Facilitate social comparison | During group sessions progress, challenges and solutions are shared |
| Stress management/emotional control training | Specific component of the educational program to reduce stress as a direct influence of glucose levels and as an indirect influence of glucose levels through diet, exercise and sleep |
| Motivational interviewing | Used during individual consultation with participants |

Behavior change techniques as described by Michie et al [64].
